# Associations of unconventional natural gas development with depression symptoms and disordered sleep in Pennsylvania

**DOI:** 10.1038/s41598-018-29747-2

**Published:** 2018-07-27

**Authors:** Joan A. Casey, Holly C. Wilcox, Annemarie G. Hirsch, Jonathan Pollak, Brian S. Schwartz

**Affiliations:** 1 Division of Environmental Health Sciences, University of California, Berkeley School of Public Health, Berkeley, USA; 2 Department of Mental Health, Johns Hopkins University Bloomberg School of Public Health, Baltimore, Maryland USA; 3 Department of Epidemiology and Health Services Research, Geisinger, Danville, Pennsylvania USA; 4 Department of Environmental Health and Engineering, Johns Hopkins Bloomberg School of Public Health, Baltimore, Maryland USA; 5 Department of Medicine, Johns Hopkins School of Medicine, Baltimore, Maryland USA

## Abstract

Environmental and community factors may influence the development or course of depression and sleep problems. We evaluated the association of unconventional natural gas development (UNGD) with depression symptoms and disordered sleep diagnoses using the Patient Health Questionnaire-8 and electronic health record data among Geisinger adult primary care patients in Pennsylvania. Participants received a retrospective metric for UNGD at their residence (very low, low, medium, and high) that incorporated dates and durations of well development, distance from patient homes to wells, and well characteristics. Analyses included 4,762 participants with no (62%), mild (23%), moderate (10%), and moderately severe or severe (5%) depression symptoms in 2014–2015 and 3,868 disordered sleep diagnoses between 2009–2015. We observed associations between living closer to more and bigger wells and depression symptoms, but not disordered sleep diagnoses in models weighted to account for sampling design and participation. High UNGD (vs. very low) was associated with depression symptoms in an adjusted negative binomial model (exponentiated coefficient = 1.18, 95% confidence interval [CI]: 1.04–1.34). High and low UNGD (vs. very low) were associated with depression symptoms (vs. none) in an adjusted multinomial logistic model. Our findings suggest that UNGD may be associated with adverse mental health in Pennsylvania.

## Introduction

Unconventional natural gas development (UNGD) is a long-lasting industrial process with potential environmental and social impacts, including noise, light, vibration, truck traffic, air, water, and soil pollution, social disruption, crime, and stress and anxiety related to these features as well as rapid industrial development^1–3^. UNGD involves pad preparation, drilling, stimulation (“fracking”), and production^1^. Operators in Pennsylvania had drilled 9,669 wells in the Marcellus shale by the end of 2015^4^ and Pennsylvania led the country in shale gas production in 2016^5^.

Growth in energy production has resulted in both local economic benefits and concern about potential health consequences. Economists have reported inconsistent effects on property values^6^ and increased employment and increased wages in counties with UNGD^7,8^, but the permanency of these benefits remains uncertain^9,10^. Public health researchers have found associations between UNGD and adverse birth outcomes^11–14^, asthma exacerbations^15^, and self-reported health problems or symptoms^16–18^, all outcomes with environmental and social risk factors. No prior epidemiologic study, however, has considered clinically-diagnosed sleep problems or a mental health outcome measured via a validated scale^19^. We considered these outcomes as important to evaluate in relation to UNGD given the biologically plausible relationship with UNGD and the significant societal costs of these outcomes. Major depressive disorder cost $210.5 billion and accounted for 3.7% of total U.S. disability-adjusted life years in 2010^20,21^, and over one-third of U.S. adults did not meet recommended sleep durations in 2014^22^.

**Figure 1:**
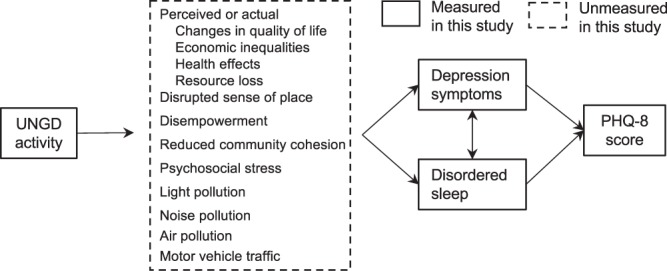
Hypothesized relationships between unconventional natural gas development (UNGD), associated physical and psychological exposures, disordered sleep and depression symptoms measured via the eight-item Patient Health Questionnaire-8 (PHQ-8) depression scale. The solid rectangle identifies factors measured in this study and the dashed rectangle identifies unmeasured factors.

Evidence suggests that depression and sleep problems may co-occur^23,24^ and that UNGD could influence these outcomes via several pathways (Fig. 1). Individuals living near UNGD have reported reduced life satisfaction, feelings of disempowerment, social stress, negative psychological states, and disruption in sense of place (i.e., meaning and attachments that residents have for their community)^25–31^. A growing body of evidence also links particulate air pollution, an environmental hazard associated with UNGD, to depression and anxiety^32,33^. Further, nighttime noise and light pollution can disrupt sleep, with potential consequences for mental health^34–37^.

Here, we evaluated the association of UNGD in the Marcellus shale in Pennsylvania with depression symptoms and disordered sleep diagnoses, measured via questionnaire and electronic health record (EHR) data, respectively. We also explored effect modification of the UNGD-depression symptoms association by antidepressant medication use under the hypothesis that those not receiving treatment may respond more strongly to UNGD exposure.

## Results

Of the 4,932 subjects in the study, 170 did not answer any PHQ-8 questions, 2,976 had no significant depression symptoms, 1,075 had mild depression symptoms, 454 had moderate depression symptoms, and 257 had moderately severe/severe depression symptoms in 2014–2015 (Table 1). Participants with more severe depression symptoms, compared to those with no or less severe symptoms, were more likely to be female, on Medical Assistance, take antidepressants, and have heavy alcohol use (all p < 0.01). We identified 8,578 disordered sleep diagnoses between January 2009 and June 2015 using EHR data among 1,699 of the 4,932 study subjects. The remaining study subjects did not have disordered sleep diagnoses using our criteria. After randomly selecting one disordered sleep diagnosis per person per year, we included 3,868 disordered sleep diagnoses over 6 years. Participants with at least one disordered sleep diagnosis, compared to those with none, were more likely to be female and to be older (both p < 0.05).

**Table 1:** Descriptive statistics by depression symptoms identified via the eight-item Patient Health Questionnaire-8 (PHQ-8) depression scale, 2014–2015.

Variable	Depression symptoms				
	0; None	1–9; Mild	9–14; Moderate	15–24; Moderately severe/severe	Missing
Total number^a^, n (%^b^)	2,976 (100)	1,075 (100)	454 (100)	257 (100)	170 (100)
UNGD^c^ metric, n (%)					
Very low	756 (25.4)	259 (24.1)	117 (25.8)	62 (24.1)	39 (22.9)
Low	726 (24.4)	285 (26.5)	113 (24.9)	66 (25.7)	43 (25.3)
Medium	776 (26.1)	253 (23.5)	101 (22.2)	58 (22.6)	45 (26.5)
High	718 (24.1)	278 (25.9)	123 (27.1)	71 (27.6)	43 (25.3)
	p^d^ = 0.65				
Race, n (%)					
White	2766 (92.9)	1005 (93.5)	420 (92.5)	228 (88.7)	158 (92.9)
Black	88 (3.0)	33 (3.1)	14 (3.1)	8 (3.1)	3 (1.8)
Hispanic	122 (4.1)	37 (3.4)	20 (4.4)	21 (8.2)	9 (5.3)
	p = 0.11				
Female, n (%)	1829 (61.5)	721 (67.1)	294 (64.8)	191 (74.3)	87 (51.2)
	p < 0.01				
Medical Assistance, n (%)	138 (4.6)	107 (10.0)	80 (17.6)	84 (32.7)	12 (7.1)
	p < 0.01				
Smoking status, n (%)					
Never	1774 (59.6)	588 (54.7)	233 (51.3)	107 (41.6)	83 (48.8)
Current	278 (9.3)	162 (15.1)	81 (17.8)	67 (26.1)	18 (10.6)
Former	924 (31.0)	325 (30.2)	140 (30.8)	83 (32.3)	69 (40.6)
	p < 0.01				
Community type, n (%)					
Borough	799 (26.8)	284 (26.4)	131 (28.9)	80 (31.1)	46 (27.1)
City	202 (6.8)	99 (9.2)	45 (9.9)	34 (13.2)	9 (5.3)
Township	1975 (66.4)	692 (64.4)	278 (61.2)	143 (55.6)	115 (67.6)
	p < 0.01				
Well water, n (%)	1129 (37.9)	410 (38.1)	147 (32.4)	66 (25.7)	75 (44.1)
	p < 0.01				
Alcohol status, n (%)					
No	1256 (42.2)	431 (40.1)	183 (40.3)	121 (47.1)	82 (48.2)
Yes, not heavy	1505 (50.6)	524 (48.7)	191 (42.1)	92 (35.8)	78 (45.9)
Yes, heavy	215 (7.2)	120 (11.2)	80 (17.6)	44 (17.1)	10 (5.9)
	p < 0.01				
On depression medication, n (%)	601 (20.2)	396 (36.8)	213 (46.9)	138 (53.7)	43 (25.3)
	p < 0.01				
Number of PHQ-8 questions missing, n (%)					
0	2796 (94)	977 (90.9)	411 (90.5)	235 (91.4)	0 (0)
1–7	180 (6)	98 (9.1)	43 (9.5)	22 (8.6)	0 (0)
All 8	0 (0)	0 (0)	0 (0)	0 (0)	170 (100)
	p < 0.01				
BMI (kg/m^2^), mean	29.6	30.5	31.5	32.1	29.4

Abbreviations: BMI = body mass index; PHQ-8 = Patient Health Questionnaire-8; UNGD = unconventional natural gas development.

^a^The follow-up responders outside of Pennsylvania (n = 34) were excluded.

^b^Column percent.

^c^The UNGD metric was a composite for four phases of well development (pad preparation, drilling, stimulation, and production) and was assigned for the two weeks prior to survey return.

^d^p-values from chi-squared tests of each covariate with the different levels of depression symptoms (no, mild, moderate, moderately severe/severe depression symptoms; missing).

By December 31, 2014, companies had drilled 8807 unconventional wells in the Pennsylvania Marcellus shale^4^. In unadjusted truncated-weighted multinomial logistic regression models, the high and low groups of the UNGD activity index (vs. very low) were associated with increased odds of mild depression symptoms (vs. none, odds ratios [95% confidence interval] = 1.49 [1.11–1.99] and 1.52 [1.13–2.04], respectively). We also observed associations between the high and low groups of the UNGD activity index (vs. very low) and mild depression symptoms (vs. none) in an adjusted multinomial logistic model (Table 2) and the number of depression symptoms (continuous variable) in an adjusted negative binomial model (Table 3). There was no significant association between the medium UNGD activity group (vs. very low) and depression symptoms in either model. In the adjusted multilevel model for the longitudinal disordered sleep outcome, UNGD was not associated with disordered sleep diagnoses between 2009–2015 (Table 4) or in analyses restricted to diagnoses in 2014–2015 (see Supplementary Table S3). We also observed no association between UNGD and encounters that resulted in both a disordered sleep diagnosis and sleep-related medication order (see Supplementary Table S4).

**Table 2:** Association of UNGD and depression symptoms identified via the eight-item Patient Health Questionnaire-8 (PHQ-8) depression scale in weighted survey multinomial logistic models (n = 4,762^a^).

UNGD group^b^	Mild depression symptoms^c,d^	Moderate depression symptoms^c,d^	Moderately severe/severe depression symptoms^c,d^
	OR (95% CI)	OR (95% CI)	OR (95% CI)
Very low	1.00	1.00	1.00
Low	1.63 (1.21–2.19)	1.22 (0.80–1.86)	1.13 (0.61–2.06)
Medium	1.25 (0.92–1.71)	1.04 (0.68–1.60)	0.89 (0.47–1.69)
High	1.51 (1.12–2.04)	1.26 (0.83–1.92)	1.39 (0.76–2.54)

Abbreviations: CI = confidence interval; OR = odds ratio; UNGD = unconventional natural gas development.

^a^Excludes the follow-up responders outside of Pennsylvania (n = 34) and those who answered no depression symptom questions (n = 170).

^b^The UNGD metric was a composite for four phases of well development (pad preparation, drilling, stimulation, and production) and was assigned for the two weeks prior to follow-up survey return.

^c^Models used truncated survey weights and adjusted for race/ethnicity (White non-Hispanic, Black non-Hispanic, Hispanic), sex (male, female), Medical Assistance (no, yes), age (years, linear and quadratic terms), smoking status (never, former, current), alcohol status (no; yes, not heavy; yes, heavy), body mass index (BMI, kg/m^2^, linear and quadratic terms), community socioeconomic deprivation (linear and quadratic terms), and water source (municipal water, well water).

^d^No depression symptoms was the base outcome.

**Table 3:** Association of UNGD and depression symptoms identified via the eight-item Patient Health Questionnaire-8 (PHQ-8) depression scale in weighted survey negative binomial models (n = 4,762^a^).

UNGD group^b^	Depression symptoms^c^
	Exponentiated coefficient^d^ (95% CI)
Very low	1.00
Low	1.14 (1.01–1.29)
Medium	1.03 (0.91–1.17)
High	1.18 (1.04–1.34)

Abbreviations: CI = confidence interval; UNGD = unconventional natural gas development.

^a^Excludes follow-up responders outside of Pennsylvania (n = 34) and those that answered no depression symptom questions (n = 170).

^b^The UNGD metric was a composite for four phases of well development (pad preparation, drilling, stimulation, and production) and was assigned for the two weeks prior to follow-up survey return.

^c^Models included truncated survey weights and adjusted for race/ethnicity (White non-Hispanic, Black non-Hispanic, Hispanic), sex (male, female), Medical Assistance (no, yes), age (years, linear and quadratic terms), smoking status (never, former, current), alcohol status (no; yes, not heavy; yes, heavy), body mass index (BMI, kg/m^2^, linear and quadratic terms), community socioeconomic deprivation (linear and quadratic terms), and water source (municipal water, well water).

^d^Ratio of mean symptom counts.

**Table 4:** Association between UNGD and disordered sleep in a weighted generalized estimating equations model (n = 3,658^a^).

UNGD group^b^	Disordered sleep diagnosis^c^
	OR (95% CI)
Very low	1.00
Low	0.96 (0.73–1.25)
Medium	1.06 (0.80–1.40)
High	1.06 (0.79–1.42)

Abbreviations: CI = confidence interval; OR = odds ratio; UNGD = unconventional natural gas development.

^a^Includes 1699 cases and frequency-matched controls. Excludes follow-up responders outside of Pennsylvania (n = 34) and those that answered no depression symptom questions (n = 170).

^b^The UNGD metric was a composite for four phases of well development (pad preparation, drilling, stimulation, and production) and was assigned for the three months prior to each event.

^c^Models included truncated survey weights and adjusted for race/ethnicity (White non-Hispanic, Black non-Hispanic, Hispanic), sex (male, female), Medical Assistance (no, yes), age (years, linear and quadratic terms), smoking status (never, former, current), alcohol status (no; yes, not heavy; yes, heavy), body mass index (BMI, kg/m^2^, linear and quadratic terms), community socioeconomic deprivation (linear and quadratic terms), and water source (municipal water, well water).

When we added a cross-product between UNGD and antidepressant medication use to the model we found no evidence of multiplicative interaction, the p-value from the Wald test of the cross-product was 0.14 and 0.12 in the multinomial logistic and negative binomial models, respectively. In the sensitivity analysis to evaluate the influence of weighting on associations, we observed stronger associations between UNGD and depression symptoms using full weights, and no association between UNGD and depression symptoms using no weights (Tables S5 and S6). Additionally, results did not change when we limited the multinomial logistic models to participants that completed all 8 questions on the PHQ-8 (Table S7).

## Discussion

In this study of nearly 5,000 adults in Pennsylvania in 2015, we observed an association between living closer to more and bigger UNGD wells and more depression symptoms as measured by the PHQ-8. Antidepressant use did not appear to act as an effect modifier. We found no evidence of an association between UNGD and disordered sleep diagnoses. To our knowledge, this study represents the first to evaluate associations between UNGD and mental health and sleep using a validated instrument and clinical diagnoses.

Our results align with prior qualitative studies finding mental distress among members of UNGD communities^19^. For example, in an analysis of letters to the editor about UNGD in a newspaper in Pennsylvania, stress was a major theme^2^, and a sample of people living near UNGD reported stress as their most common symptom^38^. In Texas, Maguire and colleagues used county-level Behavioral Risk Factor Surveillance System data and found an association between UNGD and reduced life satisfaction among women and an increased number of poor mental health days among both sexes^26^. Perceived changes in quality of life^30,39,40^, health effects^29^, or resource loss^25^, as well as feelings of disempowerment^31,41^, a disrupted sense of place^25,27^, and a loss of community cohesion^10,25^ could potentially explain our observed association between UNGD activity and depression symptoms.

In a previous study, we found an association between UNGD and nasal and sinus, migraine, and fatigue symptoms^18^. These outcomes may co-occur or lie on the pathway between UNGD and mental health outcomes and sleep disturbances^42,43^. In addition, the findings could have relevance to our prior reported associations of UNGD with asthma exacerbations^15^; as stress could be a plausible mediator between both UNGD and depression symptoms and UNGD and asthma exacerbations^44,45^.

UNGD could also influence mental health via an air, noise, or light pollution pathway. Our model incorporated all phases of UNGD (from well pad development to production), each of which releases air pollutants from truck traffic, diesel powered machinery, and fugitive emissions^1^. Short- and long-term exposure to air pollution has been associated with depression symptoms. For example, in a study in Korea that evaluated long-term exposure, a 10 µg/m^3^ increase in PM_2.5_ over the prior year was associated with 1.47 times the risk of a diagnosis of major depression disorder^33^. Air pollution may increase pro-inflammatory markers in the blood^46^ and overall oxidative stress^47^, both factors previously associated with depression^48,49^. Nighttime noise and light can affect sleep duration and quality, which in turn could lead to adverse mental health outcomes^34–37^. For example, Orban *et al*. reported an association between 24-hour traffic noise and high depressive symptoms, an association that was stronger among participants reporting insomnia at baseline^50^.

More extreme environmental exposures, like those resulting from disasters, have been associated with adverse mental health outcomes^51^. Telephone interviews after the Deepwater Horizon oil spill revealed increased depressive symptoms and mental distress among women with physical exposure to the event^52^. Oil and gas wastewater injection into class II injection wells in Oklahoma has triggered felt earthquakes over the past decade^53^. A recent study reported an association between earthquakes with magnitude greater than four and increased Google search episodes for anxiety^54^. UNGD, a human-made industry with several resulting individual and community exposures in Pennsylvania, could perhaps result in a similar mental health impacts.

This study had several strengths, in particular its large sample size. It assessed depression symptoms with a validated questionnaire and scale, a strength because EHR data may not capture depression and its symptoms well^55^. In addition, prior population-based studies have reported high sensitivity and specificity using PHQ-8 scores ≥ 10 to identify individuals with major depression^56,57^. A small portion of participants did not answer all PHQ-8 questions, but we observed no difference in results when stratifying analyses by participants with complete and incomplete questionnaires. Our study questionnaire did not mention UNGD, which should have reduced the possibility of over-reporting of symptoms among participants around higher UNGD activity (i.e., same source bias). Additionally, the UNGD metric captured the time-varying nature of well development and incorporated distance to multiple wells and size and phase of wells in the activity metric. The metric, however, did not allow us to determine which, if any, of the hypothesized pathways in Fig. 1 may account for the association between UNGD and depression symptoms.

This study had additional limitations. Exposure assignments likely included some misclassification for two reasons. First, we used patient address at the time of questionnaire mailing to assign the UNGD activity metric. Previous work has shown, however, that the Geisinger population exhibits residential stability with just 4% of the population moving >16 km from their original address over a 3-year period^11^. Second, we succeeded in geocoding at the address-level for 89.1% of the sample. Associations did not change appreciably when we restricted analyses to this population.

Responders tended to be sicker than the general population because the original survey was designed to oversample patients with nasal and sinus symptoms^18,58^. This could limit the generalizability of our results as sicker individuals may represent vulnerable populations who might more readily develop UNGD-related depression or sleep problems^59^ or exhibit a stronger response to psychosocial stressors^60^, air^61^ or noise pollution^62^. Further, individual factors including age, race/ethnicity, sex, and underlying health may affect survey response^63^. While we used survey weights to account for the survey design and non-response, differences may still have existed between the weighted population and the source population. A previous study of pediatric patients suggested that the use of *International Classification of Diseases, 9th Revision* (*ICD-9*) codes to identify sleep disorders likely underestimates the true prevalence^64^. Our use of *ICD-9* codes and medication orders to identify these conditions likely also led to under-ascertainment. Participants may have treated disordered sleep over-the-counter; only a portion of individuals with sleep problems seek medical attention and only a subset of care-seekers receives a diagnosis or medication^65^. Future studies could consider identifying disordered sleep in clinical notes or by questionnaire^66^. We lacked biologic measures of stress (e.g., cortisol) as well as information on survey responders’ attitudes about their community generally or about UNGD specifically, factors that could have influenced their psychological and physical response to the development^25,27,67^. We did not know if survey responders had signed a lease with a drilling company. Leaseholders receive economic benefit from UNGD, making them more supportive of UNGD and possibly less likely to experience adverse psychological outcomes as a result^68^. Lastly, we did not make air quality, light, vibration, traffic, or noise level measurements, so cannot evaluate which, if any, environmental hazards were present at higher levels in the highest UNGD activity group.

In conclusion, we combined information from a mailed questionnaire, EHR data, and a time-varying measure of residential proximity to more and bigger UNGD wells to conduct the most comprehensive study to date on the potential mental health and sleep consequences of UNGD. We found an association between UNGD and depression symptoms but not with disordered sleep diagnoses. Individuals require access to mental health services with clinicians trained to screen, monitor, and treat psychological problems among populations potentially affected by UNGD^19,69^. At the same time, further research is required to disentangle the multifactorial pathways through which UNGD may influence mental health. Our findings should be interpreted in the context of prior reports of associations of UNGD with other health outcomes and suggest the need to incorporate potential mental health consequences of UNGD in risk-benefit calculations.

## Methods

### Study area

We conducted this study among Geisinger’s adult patients^58^, located in over 40 counties in central and northeast Pennsylvania in a region with a range of UNGD activity. Geisinger’s primary care population is representative of the general population of the region based on distributions of age, sex, and race/ethnicity^11^. Geisinger has had a fully-operational EHR installed since 2005. All Geisinger patients had the option to opt out of all research, but less than 0.1% did so at the time of the study. Those that did not opt out were informed that their EHR data could be used for research.

### Survey design and study population

Our study population came from a cohort originally designed to study the epidemiology of chronic rhinosinusitis and related nasal and sinus symptoms^18,58^. The survey design oversampled racial/ethnic minorities and people more likely to have nasal and sinus symptoms. In April 2014, a baseline questionnaire, a cover letter that explained the study, and a $1 bill as incentive was sent to 23,700 adults 18 years of age and older. The cover letter explained that study participation was voluntary and that if participants did not return the questionnaire they would not be included; by returning the questionnaire participants provided informed consent. Of the 23,700 letters sent, 7,847 participants responded (response rate = 33.1%)^58^. Six months later, a follow-up questionnaire, which included the eight-item Patient Health Questionnaire-8 (PHQ-8), was sent to all responders of the baseline questionnaire, of whom 4,966 responded (secondary response rate = 63.3%). Follow-up questionnaires were received from November 2014 to May 2015 (median of November 12, 2014). Using previously described methods^70^, we geocoded study subjects to their residential address listed in the EHR, 89.1% to street address, 3.1% to ZIP + 4, and 7.7% to ZIP code centroid. After excluding respondents living outside Pennsylvania (n = 34), the analysis consisted of 4,932 participants. All study protocols were reviewed and carried out in accordance with guidelines approved by the Geisinger Institutional Review Board.

### Outcome ascertainment

#### Depression symptoms

The PHQ-8 asked individuals to report symptoms in the prior two weeks, for example, “how often have you been bothered by feeling down, depressed, hopeless?”^56^. Each question on the PHQ-8 has response options: “not at all”, “several days”, “more than half the days”, or “nearly every day”, scored as 0–3 respectively. Over 90% of participants answered all eight questions, for whom we calculated their total score by summing scores from the eight questions^57^. For participants who answered fewer than eight questions, we calculated their total score as a pro-rated sum using the formula: (sum of answered questions × 8)/(number of questions answered). Of participants who answered 1–7 questions, 81% answered 7 questions. We defined current depression symptoms using the total PHQ-8 score based on previously established categories^56^, but combined the two most severe groups because few participants had a “severe” total score. Scores were categorized into 0 to <5, no significant depression symptoms; 5 to <10, mild depression symptoms; 10 to <15, moderate depression symptoms; and 15 to 24, moderately severe/severe depression symptoms^57^. We excluded participants who did not answer any PHQ-8 questions (n = 170) from statistical analyses.

#### Disordered sleep diagnoses

Disordered sleep diagnoses (case-events) among the study population were identified in Geisinger’s EHR from January 2009 to June 2015. We identified encounters (98% outpatient) in the EHR that were accompanied by *ICD-9* codes for disordered sleep (see Supplementary Table S1). We also identified orders for disordered sleep medications, using drug class “hypnotics” as well as drug subclass and name. We included all medications in the drug subclass antihistamine hypnotics, selective melatonin receptor agonists, hypnotics – tricyclic agents, and orexin receptor antagonists. In the subclass non-barbiturate hypnotics, we included all medications except midazolam hydrochloride, which is more often used for procedural sedation. We considered either an appropriate medication order or an encounter with a disordered sleep *ICD-9* code as a disordered sleep outcome. We only retained disordered sleep diagnoses from when the participant was 18 years of age or older and randomly selected one disordered sleep diagnosis per participant per year so that study subjects with many encounters for sleep disorders would not unduly contribute (see Supplementary Figure S1).

For control dates, we identified all their dates of contact with the health system, excluded contact dates within one year of a disordered sleep diagnosis, and randomly selected one encounter date per year per participant. Control dates were frequency-matched to cases on age category (i.e., 18–44, 45–61, 62–74, 75+ years), sex, and year. We used encounter dates, rather than patients, to match as controls because of the time-varying nature of UNGD and many covariates.

### Well data and activity metric assignment

We compiled well data from the Pennsylvania Department of Environmental Protection, the Pennsylvania Department of Conservation and Natural Resources, and SkyTruth, as described previously^11,15,18^. These data, collected for all unconventional natural gas wells in Pennsylvania from 2005–2015, included: latitude and longitude; dates of well pad construction, drilling, stimulation, and production; total well depth; and volume of natural gas produced biannually or annually.

We assigned UNGD activity for the four phases of well development (pad preparation, drilling, stimulation, and production) to each study subject (in the depression symptom analysis) or index date (in the disordered sleep analysis) using metrics that incorporated distances from participant residence to wells, and the density and size of wells, as in prior studies^11,15,18^. The metric has the potential to incorporate a variety UNGD-related hazards that exist on different temporal and spatial scales (e.g., regional air pollutants, local noise, truck traffic, activities that may lead to stress)^71^. We calculated the metric for each phase of well development:

$${\rm{Metric}}\,{\rm{for}}\,{\rm{participant}}\,{\rm{j}}=\sum _{t=t}^{d}\,\sum _{i=1}^{n}\frac{{s}_{i}}{{m}_{ij}^{2}}$$

where d was the date of return of the questionnaire, n was the number of wells in the given phase, *m*_*ij*_^2^ was the squared-distance (meters) between well *i* and participant *j*, and s_*i*_ was 1 for the pad production and drilling phases, total well depth (meters) of well *i* for the stimulation phase, and daily natural gas production volume (m^3^) of well *i* for the production phase. For the depression symptom analysis, for each phase of development, the metric was summed for the 14 days prior (t = d − 14) to the date of the returned follow-up questionnaire (d). We chose 14 days prior to the survey return because the PHQ-8 ascertained depression symptoms over the past two weeks. For the disordered sleep analysis, we summed the UNGD activity for the three months prior to the date of the sleep disorder diagnosis (t = d − 90). In both analyses, we z-transformed the activity metrics for each of the four phases of development, summed the transformed values, and calculated quartiles of the sums to create a composite UNGD metric that represented very low, low, medium, and high residential proximity to more and bigger UNGD wells.

### Covariates

Using the EHR and questionnaire, we created covariates for potential confounding variables: race/ethnicity; sex; Medical Assistance (a means-tested program used as a surrogate for family socioeconomic status)^72^; age at time of questionnaire return, disordered sleep diagnosis, or control date; smoking and alcohol use status; and body mass index. Antidepressant medication use in the month prior to survey return was ascertained with medication orders based on drug group (e.g., antidepressants), class (e.g., selective serotonin reuptake inhibitors [SSRIs]), sub-class, and name. We evaluated antidepressant use as an effect modifier; we hypothesized that antidepressant medication could attenuate associations of UNGD with depression symptoms. Time-varying-covariates (all but race/ethnicity and sex) were assigned before the date of questionnaire return (for the depression symptom analysis) or before the disordered sleep diagnosis or comparison date (for the disordered sleep analysis). Based on the participants’ geocoded coordinates, we assigned them to a community using a mixed definition of place (township, borough, or census tract in cities)^70^. For each community, we used the 2006–2010 American Community Survey to calculate community socioeconomic deprivation^73^. We used public water supplier service areas from the Pennsylvania Department of Environmental Protection to assign residential water source (municipal water or ground water)^74^. Patients residing in homes outside the public water supplier service area were assumed to use ground water.

### Statistical analysis

We employed sampling weights to estimate unbiased measures of association while accounting for the survey stratified sampling design, the response rate to the baseline questionnaire, and loss to follow-up from the baseline to the follow-up questionnaires (see Supplementary Table S2). Because one weight was much larger than the others, we truncated the largest weight to the next largest for our primary analyses^75^.

To build models, we first included the UNGD variable representing residential proximity to more and bigger wells (quartiles: very low, low, medium, high), and then added potential confounding variables identified *a priori*: race/ethnicity (non-Hispanic White, non-Hispanic Black, Hispanic), sex (male, female), Medical Assistance (no, yes), age (years), smoking status (never, former, current), alcohol use status (heavy [based on the Centers for Disease Control definition of heavy drinking as 8 or more drinks per week for females and 15 or more drinks for males^76^]; vs. not heavy [which included no alcohol use]), body mass index from the EHR at the visit closest to questionnaire return (BMI, kg/m^2^), community socioeconomic deprivation, and water source (municipal water, well water). We centered the continuous covariates (age, BMI, and community socioeconomic deprivation) and included them as linear and quadratic terms to allow for non-linearity. We did not include community type (i.e., township, borough, or city) in final models because it may lie on the causal pathway between UNGD and sleep and mental health. We used a 2-sided type 1 error rate of 0.05 for significance testing and Stata version 11.2 (StataCorp Inc.) and R version 3.2.2 (R Foundation for Statistical Computing) for analyses.

We fit multinomial logistic models to estimate the association UNGD with each level of depression symptoms (mild, moderate, moderately severe/severe) compared to no depression symptoms (reference outcome). We also evaluated the association of UNGD with depression symptoms using negative binomial regression, which treated the PHQ-8 score as a continuous outcome, allowing us to evaluate associations between UNGD and the continuous burden of depression symptoms, rather than with the screening tool’s categories^77^. To assess the association of proximity to more and bigger UNGD wells with disordered sleep diagnoses, we fit a survey-weighted generalized estimating equations model, to account for multiple diagnoses within participants. Because sleep diagnoses spanned 2009–2015 but most UNGD began after 2010 and attitudes towards UNGD may have changed over time, in a secondary analysis we restricted the sleep analysis to diagnoses between January 2014 and June 2015. To test a definition with a higher positive predicative value for sleep disorder, we also ran a model where a case had to have both a diagnostic code for a sleep disorder and a sleep-related medication order.

We hypothesized reduced susceptibility to stressors like UNGD among participants taking antidepressants^78,79^. To test this, we evaluated effect modification by antidepressant use by including cross-products of the UNGD variables and antidepressant medication use to our final depression symptom models. We used a Wald test to evaluate the significance of the cross-products.

In a sensitivity analysis, we evaluated the influence of our sample weights by examining associations of UNGD with depression symptoms among all subjects using the final multinomial logistic and negative binomial models without weights and with full and truncated weights. In theory, weighted models will provide less precise, but more unbiased estimates than unweighted models^80^, our rationale for using truncated weighted models as the primary analysis.

### Data Availability

Data on unconventional natural gas development in Pennsylvania are publicly available from the Pennsylvania Department of Environmental Protection, the Pennsylvania Department of Conservation and Natural Resources at http://www.dcnr.state.pa.us/topogeo/econresource/oilandgas/resrefs/wis_home/. The health data that were used in this study are protected health information and subject to many restrictions. Data may be available from the corresponding author upon reasonable request and with specific required agreements in place.

## Electronic supplementary material

Supplementary Information 
