# Supplementary material for: Associations of unconventional natural gas development with depression symptoms and disordered sleep in Pennsylvania
Source: Sci Rep. 2018 Jul 27;8:11375. doi: 10.1038/s41598-018-29747-2 (PMC6063969; doi:10.1038/s41598-018-29747-2)

Supplemental Material for "Associations of unconventional natural gas development with depression symptoms and disordered sleep in Pennsylvania"

Joan A. Casey, PhD[1]; Holly C. Wilcox, PhD[2]; Annemarie G. Hirsch, PhD[3]; Jonathan Pollak, MPP[4]; Brian S. Schwartz, MD, MS[3,4,5]

[1]Division of Environmental Health Sciences, University of California Berkeley School of Public Health, Berkeley, USA; [2]Department of Mental Health, Johns Hopkins University Bloomberg School of Public Health, Baltimore, Maryland, USA; [3]Department of Epidemiology and Health Services Research, Geisinger, Danville, Pennsylvania, USA; [4]Department of Environmental Health and Engineering, Johns Hopkins Bloomberg School of Public Health, Baltimore, Maryland, USA; [5]Department of Medicine, Johns Hopkins School of Medicine, Baltimore, Maryland, USA

Table of Contents:

Table S1. *ICD-9* codes used to identify disordered sleep.

Table S2. Calculation of sample weights.

Table S3. Association of UNGD and depression symptoms in survey multinomial logistic models using varying survey weights.

Table S4. Association between UNGD and disordered sleep in a weighted generalized estimating equations model, restricted to 2014-2015 diagnoses.

Table S5. Association between UNGD and disordered sleep (identified via a diagnostic code and sleep-related medication order) in a weighted generalized estimating equations model.

Table S6. Association of UNGD and depression symptoms in survey negative binomial models using varying survey weights.

Table S7. Association of UNGD and depression symptoms in survey multinomial logistic models: overall and among participants that answered all PHW-8 questions.

Figure S1. Flow diagram for identification of disordered sleep diagnoses.

Table S1. *ICD-9* codes used to identify disordered sleep.

ICD-9 code	Description
780.52	Insomnia
780.50	Sleep disturbance, unspecified
307.47	Other dysfunctions of sleep stages or arousal from sleep
780.59	Other sleep disturbances
307.42	Persistent disorder of initiating or maintaining sleep
780.5	Sleep disturbances
307.41	Transient disorder of initiating or maintaining sleep
307.40	Nonorganic sleep disorder, unspecified
307.48	Repetitive intrusions of sleep
780.56	Dysfunctions associated with sleep stages or arousal from sleep
780.55	Disruptions of 24-hour sleep-wake cycle

Abbreviation: *ICD-9 = International Classification of Diseases, 9th Revision, Clinical Modification*

Table S2. Calculation of sample weights.

Race/ethnicity	Higher likelihood of CRS	Intermediate likelihood of CRS	Lower likelihood of CRS
White non-Hispanic			
Identified using EHR	13,132	47,892	131,366
Received baseline survey	12,209	4,224	2,775
Responded to baseline survey	4,691	1,481	871
Responded to follow-up survey	3,076	950	551
Sample weight	4.27	50.41	238.41
Black non-Hispanic			
Identified using EHR	170	991	2,832
Received baseline survey	159	903	1,109
Responded to baseline survey	35	144	155
Responded to follow-up survey	17	67	62
Sample weight	10.00	14.79	45.68
Hispanic			
Identified using EHR	192	1,035	3,159
Received baseline survey	181	966	1,174
Responded to baseline survey	35	206	167
Responded to follow-up survey	19	92	98
Sample weight	10.11	11.25	32.23

Abbreviations: CRS = chronic rhinosinusitis; EHR = electronic health record

Table S3. Association between UNGD and disordered sleep in a weighted generalized estimating equations model, restricted to 2014-2015 diagnoses (n = 1,705[a]).

UNGD group[b]	Disordered sleep diagnosis[c] OR (95% CI)
Very low	1.00
Low	1.23 (0.64 - 2.35)
Medium	1.42 (0.74 - 2.73)
High	1.28 (0.67 - 2.42)

Abbreviations: CI = confidence interval; OR = odds ratio; UNGD = unconventional natural gas development

[a] Includes 775 cases and frequency-matched controls. Excludes follow-up responders outside of Pennsylvania (n = 34) and those that answered no depression symptom questions (n = 170).

[b] The UNGD metric was a composite for four phases of well development (pad preparation, drilling, stimulation, and production) and was assigned for the three months prior to each event.

[c] Models included truncated survey weights and adjusted for race/ethnicity (White non-Hispanic, Black non-Hispanic, Hispanic), sex (male, female), Medical Assistance (no, yes), age (years, linear and quadratic terms), smoking status (never, former, current), alcohol status (no; yes, not heavy; yes, heavy), body mass index (BMI, kg/m^2, linear and quadratic terms), community socioeconomic deprivation (linear and quadratic terms), and water source (municipal water, well water).

Table S4. Association between UNGD and disordered sleep (identified via a diagnostic code and sleep-related medication order) in a weighted generalized estimating equations model, (n = 2,006[a]).

UNGD group[b]	Disordered sleep diagnosis + medication
	OR (95% CI)
Very low	1.00
Low	1.02 (0.89 – 1.17)
Medium	1.14 (0.98 – 1.32)
High	1.12 (0.94 – 1.33)

Abbreviations: CI = confidence interval; OR = odds ratio; UNGD = unconventional natural gas development

[a] Includes 693 cases and frequency-matched controls. Excludes follow-up responders outside of Pennsylvania (n = 34) and those that answered no depression symptom questions (n = 170).

[b] The UNGD metric was a composite for four phases of well development (pad preparation, drilling, stimulation, and production) and was assigned for the three months prior to each event.

[c] Models included truncated survey weights and adjusted for race/ethnicity (White non-Hispanic, Black non-Hispanic, Hispanic), sex (male, female), Medical Assistance (no, yes), age (years, linear and quadratic terms), smoking status (never, former, current), alcohol status (no; yes, not heavy; yes, heavy), body mass index (BMI, kg/m^2, linear and quadratic terms), community socioeconomic deprivation (linear and quadratic terms), and water source (municipal water, well water).

Table S5. Association of UNGD and depression symptoms in survey multinomial logistic models using varying survey weights (n=4,762[a]).

UNGD group[b]	Mild depression symptoms[c,d] OR (95% CI)	Moderate depression symptoms[c,d] OR (95% CI)	Moderately severe/severe depression symptoms[c,d] OR (95% CI)
Truncated survey weights[e]			
Very low	1.00	1.00	1.00
Low	1.63 (1.21 - 2.19)	1.22 (0.80 - 1.86)	1.13 (0.61 - 2.06)
Medium	1.25 (0.92 - 1.71)	1.04 (0.68 - 1.60)	0.89 (0.47 - 1.69)
High	1.51 (1.12 - 2.04)	1.26 (0.83 - 1.92)	1.39 (0.76 - 2.54)
Full survey weights			
Very low	1.00	1.00	1.00
Low	1.72 (1.14 - 2.59)	1.20 (0.67 - 2.14)	0.93 (0.37 - 2.34)
Medium	1.29 (0.84 - 1.98)	1.23 (0.66 - 2.28)	0.68 (0.26 - 1.79)
High	1.95 (1.28 - 2.97)	1.77 (0.98 - 3.20)	1.47 (0.63 - 3.46)
No survey weights			
Very low	1.00	1.00	1.00
Low	1.23 (1.003 - 1.50)	1.04 (0.78 - 1.39)	1.19 (0.82 - 1.74)
Medium	0.996 (0.81 - 1.22)	0.84 (0.63 - 1.13)	0.91 (0.61 - 1.34)
High	1.12 (0.92 - 1.37)	1.06 (0.80 - 1.40)	1.11 (0.76 - 1.61)

Abbreviations: CI = confidence interval; OR = odds ratio; UNGD = unconventional natural gas development

[a] Excludes follow-up responders outside of Pennsylvania (n = 34) and those who answered no depression symptom questions (n = 170).

[b] The UNGD metric was a composite for four phases of well development (pad preparation, drilling, stimulation, and production) and was assigned for the two weeks prior to follow-up survey return.

[c] Models adjusted for race/ethnicity (white non-Hispanic, black non-Hispanic, Hispanic), sex (male, female), Medical Assistance (no, yes), age (years, linear and quadratic terms), smoking status (never, former, current), alcohol status (no; yes, not heavy; yes, heavy), body mass index (BMI, kg/m, linear and quadratic terms), community socioeconomic deprivation (linear and quadratic terms), and water source (municipal water, well water).

[d] No depression symptoms was the base outcome.

[e] Primary analysis

Table S6. Association of UNGD and depression symptoms in survey negative binomial models (n=4,762[a]) using varying survey weights.

UNGD group[b]	Depression symptoms[c] Exponentiated coefficient[d] (95% CI)
Truncated survey weights[e]	
Very low	1.00
Low	1.14 (1.01 - 1.29)
Medium	1.03 (0.91 - 1.17)
High	1.18 (1.04 - 1.34)
Full survey weights	
Very low	1.00
Low	1.12 (0.94 - 1.34)
Medium	1.07 (0.88 - 1.29)
High	1.29 (1.08 - 1.56)
No survey weights	
Very low	1.00
Low	1.05 (0.96 - 1.15)
Medium	0.96 (0.88 - 1.05)
High	1.03 (0.94 - 1.13)

Abbreviations: CI = confidence interval; UNGD = unconventional natural gas development

[a] Excludes follow-up responders outside of Pennsylvania (n = 34) and those that answered no depression symptom questions (n = 170).

[b] The UNGD metric was a composite for four phases of well development (pad preparation, drilling, stimulation, and production) and was assigned for the two weeks prior to follow-up survey return.

[c] Models adjusted for race/ethnicity (White non-Hispanic, Black non-Hispanic, Hispanic), sex (male, female), Medical Assistance (no, yes), age (years, linear and quadratic terms), smoking status (never, former, current), alcohol status (no; yes, not heavy; yes, heavy), body mass index (BMI, kg/m, linear and quadratic terms), community socioeconomic deprivation (linear and quadratic terms), and water source (municipal water, well water).

[d] Ratio of mean symptom counts

[e] Primary analysis

Table S7. Association of UNGD and depression symptoms in survey multinomial logistic models: overall (n = 4,762[a]) and among participants that answered all PHW-8 questions

UNGD group[b]	Mild depression symptoms[c,d]	Moderate depression symptoms[c,d]	Moderately severe/severe depression symptoms[c,d]
	OR (95% CI)	OR (95% CI)	OR (95% CI)
**Overall (n = 4,762[a])**			
Very low	1.00	1.00	1.00
Low	1.63 (1.21 - 2.19)	1.22 (0.80 - 1.86)	1.13 (0.61 - 2.06)
Medium	1.25 (0.92 - 1.71)	1.04 (0.68 - 1.60)	0.89 (0.47 - 1.69)
High	1.51 (1.12 - 2.04)	1.26 (0.83 - 1.92)	1.39 (0.76 - 2.54)
**Answered all 8 questions (n = 4,419)**			
Very low	1.00	1.00	1.00
Low	1.65 (1.21 - 2.22)	1.06 (0.68 - 1.64)	1.27 (0.69 - 2.36)
Medium	1.33 (0.96 - 1.84)	0.95 (0.61 - 1.50)	0.83 (0.42 - 1.65)
High	1.61 (1.12 - 2.24)	1.24 (0.79 - 1.94)	1.34 (0.70 - 2.58)

Abbreviations: CI = confidence interval; OR = odds ratio; PHQ-8 = Patient Health Questionnaire-8; UNGD = unconventional natural gas development

[a] Excludes the follow-up responders outside of Pennsylvania (n = 34) and those who answered no depression symptom questions (n = 170).

[b] The UNGD metric was a composite for four phases of well development (pad preparation, drilling, stimulation, and production) and was assigned for the two weeks prior to follow-up survey return.

[c] Models used truncated survey weights and adjusted for race/ethnicity (White non-Hispanic, Black non-Hispanic, Hispanic), sex (male, female), Medical Assistance (no, yes), age (years, linear and quadratic terms), smoking status (never, former, current), alcohol status (no; yes, not heavy; yes, heavy), body mass index (BMI, kg/m^2, linear and quadratic terms), community socioeconomic deprivation (linear and quadratic terms), and water source (municipal water, well water).

[d] No depression symptoms was the base outcome.

Figure S1. Flow diagram for identification of disordered sleep diagnoses. Disordered sleep events were identified using medications and *ICD-9* codes from encounters (98% outpatient). In this figure, the numbers refer to counts of medications for disordered sleep and encounters with *ICD-9* codes for disordered sleep. Abbreviation: *ICD-9 = International Classification of Diseases, 9th Revision, Clinical Modification*

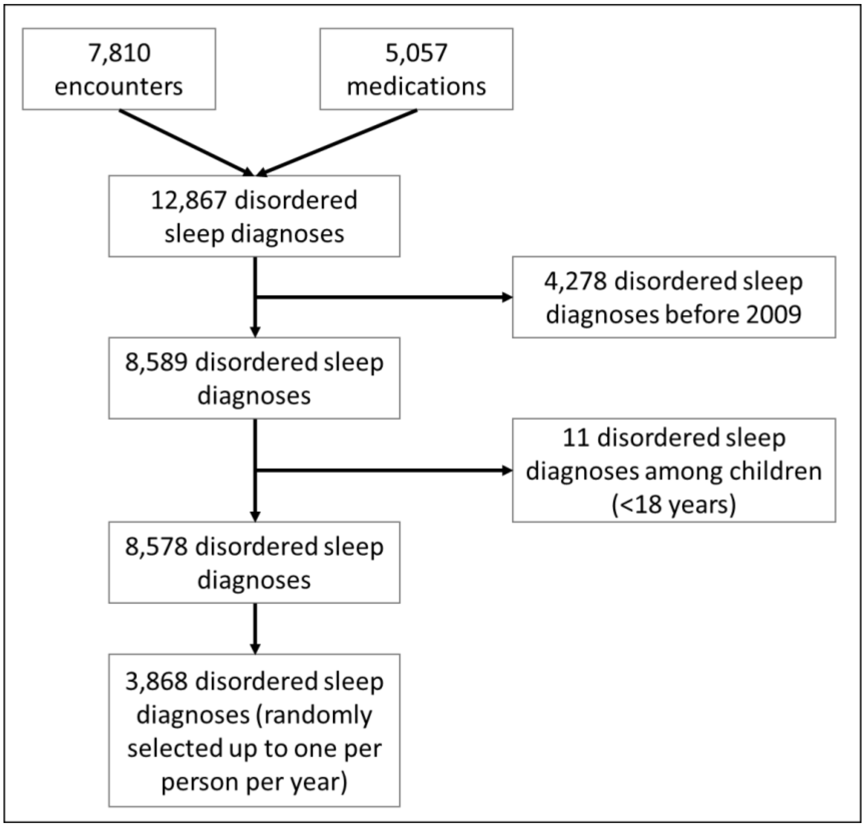

Supplement: Supplementary file 1 — Supplementary Information [file 41598_2018_29747_MOESM1_ESM.pdf]
